# Targeting Autophagy to Counteract Obesity-Associated Oxidative Stress

**DOI:** 10.3390/antiox10010102

**Published:** 2021-01-12

**Authors:** Federico Pietrocola, José Manuel Bravo-San Pedro

**Affiliations:** 1 Department of Bioscience and Nutrition, Karolinska Institute, Neo Blickagången 16, 14183 Huddinge, Sweden; 2 Departamento de Fisiología, Facultad de Medicina, Universidad Complutense de Madrid, Plaza Ramón y Cajal s/n, Ciudad Universitaria, 28040 Madrid, Spain

**Keywords:** obesity, stress, autophagy, antioxidants

## Abstract

Reactive oxygen species (ROS) operate as key regulators of cellular homeostasis within a physiological range of concentrations, yet they turn into cytotoxic entities when their levels exceed a threshold limit. Accordingly, ROS are an important etiological cue for obesity, which in turn represents a major risk factor for multiple diseases, including diabetes, cardiovascular disorders, non-alcoholic fatty liver disease, and cancer. Therefore, the implementation of novel therapeutic strategies to improve the obese phenotype by targeting oxidative stress is of great interest for the scientific community. To this end, it is of high importance to shed light on the mechanisms through which cells curtail ROS production or limit their toxic effects, in order to harness them in anti-obesity therapy. In this review, we specifically discuss the role of autophagy in redox biology, focusing on its implication in the pathogenesis of obesity. Because autophagy is specifically triggered in response to redox imbalance as a quintessential cytoprotective mechanism, maneuvers based on the activation of autophagy hold promises of efficacy for the prevention and treatment of obesity and obesity-related morbidities.

## 1. Introduction

### 1.1. Obesity 

Obesity is a medical condition defined by an excessive amount of body fat, which leads to a progressive deterioration of the general health status [1]. The penetrance of obesity within the general population is drastically ramping up, highlighting this metabolic disorder as a major health problem worldwide. According to the guidelines of the World Health Organization, “overweight” in adults is defined as a condition suffered by individuals with a body mass index (BMI) between 25 and 30 kg/m^2^ (30–70% of the population within European Union), while obesity manifests when the subjects reach a BMI > 30 kg/m^2^ (10–30% of the adult population within European Union) [2]. Excess body fat underlies the appearance of both short- and long-term health problems, as it is directly associated with various forms of physical disability and psychological discomfort, while dramatically heightening the risk of premature death, diabetes, metabolic and cardiovascular disorders, chronic inflammatory diseases such as psoriasis, and malignant transformation [3]. The elevated burden of direct or indirect medical costs related to obesity, encompassing prevention, diagnosis, and treatment services, as well as decreased work productivity and increased disability or premature death, are estimated to severely impact on the global healthcare system [4]. 

Obesity clinically manifests as consequence of an over sustained positive energy balance, in turn linked to settings of low energy expenditure (e.g., sedentarism) and/or elevated calorie uptake prolonged over time. Conversely, non-pharmacological approaches based on the reduction of calorie intake from nutritional sources or on the enhanced oxidation of anabolic substrates (e.g., physical activity) are effective in correcting this energetic imbalance and can therefore be considered as valuable preventative or curative measures for limiting the obese phenotype. Although aberrant lifestyle behaviors account for the large majority of the cases of obesity reported worldwide, it is worth noting that additional genetic, environmental, or iatrogenic factors, as well as concurrent pathologies, might account for the appearance of pathologically meaningful excess body fat in subjects carrying a healthy routine [5].

The excessive accumulation of adipose tissue within typical fat deposits or atypical anatomical sites culminates into systemic lipotoxicity, which is in turn associated with events of tissue damage [6]. Because lipids are essential components of biological membranes, dysregulated fat metabolism affects the biophysical properties (and the composition) of cellular lipid bilayers, resulting into organelle damage and exacerbated cellular stress. In line with this notion, altered lipid metabolism associated with the obesity condition underlies (among others) (i) the disruption of calcium homeostasis, resulting into Endoplasmic Reticulum (ER) stress [7]; (ii) the maladaptive accumulation of unfolded proteins, which enhances proteotoxic stress and fosters aberrant lipogenesis [8]; and (iii) the formation of highly reactive lipids species that can damage biomolecules (such as membrane-bound mitochondrial enzymes and DNA), thereby amplifying oxidative stress [9]. In order to cope with this lipotoxic environment, cells have elaborated adequate countermeasures to maintain cellular and organismal homeostasis, being autophagy a quintessential mechanism of defense against lipotoxic cues [10]. Through autophagy, cells can normalize intracellular fat content by breaking down lipid droplets (‘lipophagy’) [11], recycle part of the damaged ER (through ER-phagy, to restrain ER stress), or dysfunctional mitochondria [12] (through mitophagy, to dampen the detrimental production of reactive oxygen species (ROS) in pathological conditions associated with obesity). 

### 1.2. Oxidative Stress

Oxidative stress occurs as consequence of an imbalance between ROS production and cellular antioxidant defenses. ROS are hyper-reactive entities that form when oxygen accepts an electron; the reduction of oxygen leaves the electron in an unpaired orbital, thereby endowing radical oxygen with the capacity to react with other biomolecules, including DNA [13]. ROS are inherent to the homeostatic activity of cells, as they constitute the main metabolic byproduct of cellular aerobic activity. Accordingly, oscillations in the levels of ROS that occur within the physiological boundaries are central for the regulation of several cellular processes including proliferation, differentiation and signaling. Nonetheless, the supraphysiological production of ROS, paralleled by a reduction in the antioxidant buffering capacity of the cell, drastically elevates the levels of free radicals and therefore leads to cellular damage. At the systemic level, ROS overproduction associated with nutritional unbalance is among the major determinant of the general deterioration of the health status tied to obesity [14].

Free-radical-induced oxidative damage has been reportedly involved in the pathogenesis of several diseases, such as obesity, diabetes mellitus, neurodegenerative disorders, cardiovascular and respiratory diseases, cataracts, rheumatoid arthritis, and some types of cancer [15]. In view of their broad impact on human health, it is primordial to shed light on the main intracellular sources of ROS and to elucidate the mechanisms through which cells sustain the increased burden of oxidative stress. 

Several organelles display the capacity to generate ROS, including lysosomes, peroxisomes, and endoplasmic reticulum, yet most intracellular ROS production is estimated to occur within mitochondria. At the mitochondrial level, superoxide radicals are produced in the complexes I and III of the electron transport chain upon transfer of electrons to molecular oxygen [16]. Superoxide accounts for a large fraction of ROS generated intracellularly and acts as a precursor of most other ROS of biological importance: H_2_O_2_, OH^−^, peroxyl radical (HO_2_), and singlet or individual oxygen (^1^O_2_) [15]. Besides organelles, other important intracellular sources of ROS are specialized enzymes, including NADPH oxidase (NOX, a membrane-bound enzyme complex that transfers electrons from NADPH to oxygen), nitric oxide synthetase (NOS, a generator of nitric oxide from L-arginine, NADPH and O_2_), or the xanthine oxidase (XO, which catalyzes the oxidation of hypoxanthine to xanthine and uric acid) [15]. 

As mentioned above, the fine-tuning of the intracellular amount of these reactive molecules is of vital importance to the cell. Accordingly, cells have elaborated multilayered antioxidant defense systems to tightly control ROS concentration, including the following:

(1) Enzymes that degrade (or detoxifies) ROS at different levels, such as glutathione peroxidases, catalases, superoxide dismutases, peroxyredoxins, glutathione and thioredoxin reductases, hemoxygenase 1, or cytochrome c oxidase [17];
(2) Enzymes responsible for the repair of oxidized proteins, such as methionine sulfoxide reductase and xanthine oxidase [18];
(3) Regulators of the electron transport chain, such as cytochrome c oxidase complex [19];
(4) Exogenous antioxidants, including vitamins, carotenoids, polyphenols, and trace elements, such as selenium and zinc [20].

## 2. Obesity-Related Oxidative Stress

**Figure 1:**
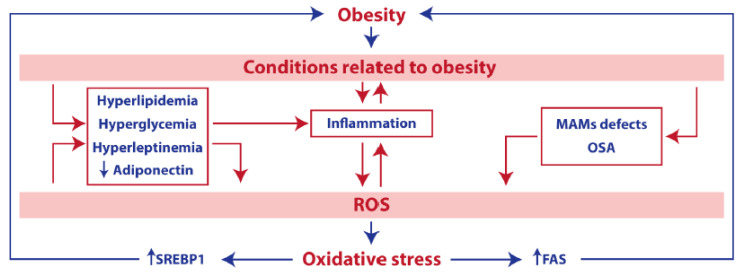
Obesity and oxidative stress. Cell intrinsic and cell extrinsic factors related to obesity, including inflammation, hyperglycemia, hyperleptinemia, hyperlipidemia, reduced adiponectin levels, obstructive sleep apnea (OSA), or mitochondria-associated Endoplasmic Reticulum (ER) membranes (MAMs) defects, can exacerbate the production of reactive oxygen species (ROS). ROS account for the induction of a detrimental lipogenic response (dependent on SREBP1 and fatty acid synthase (FAS)), which contributes to, or worsens, the obese phenotype.

Numerous studies support the observation that an elevated oxidative damage is a distinctive feature of obesity and its associated pathologies (Figure 1) [21]. Adipose tissue is composed of parenchymal (adipocytes) and stromal cells (including fibroblasts, endothelial, or immune cells), which directly contribute to its functionality. In the recent past, it has become progressively more evident that adipose tissue does not simply act as a passive energy store; it also exhibits active functions of endocrine regulation. In keeping with this notion, the adipose tissue directly participates in the systemic regulation of energy homeostasis through the secretion of adipokines or adipocytokines [22]. Importantly, alterations in the function (or the cellular composition) of the adipose tissue culminate into the heightened secretion of pro-inflammatory cytokines and are hence intrinsically tied to phenomena of low-grade chronic inflammation. In a similar vein, obesity and its related morbidities shift the phenotype of adipose tissue—resident or infiltrating macrophages from anti-inflammatory (or M2, which predominates in healthy settings) to pro-inflammatory (or M1) [23]. In keeping with this notion, reduced adiponectin secretion (a remarkable feature of the obese condition) can worsen obesity by directly mediating pro-inflammatory effects [24]. Importantly, systemic inflammation feeds the obesity–oxidative stress cycle by further increasing ROS production, while dampening antioxidants’ defense barriers.

Besides inflammation, several other conditions related to obesity have a direct impact on ROS production. Hyperlipidemia, a condition consisting in elevated levels of fats (e.g., triglycerides and cholesterol) in blood, has been thoroughly associated with both obesity and accrued oxidative damage [25]. For example, the reported increase in hepatic cholesterol levels impairs oxidative phosphorylation and perturbs mitochondrial membrane biophysical properties, ultimately hindering the correct assembly of mitochondrial respiratory supercomplexes [26]. High blood glucose (hyperglycemia), another parameter typically associated with obesity, is able to trigger the overproduction of ROS through the direct activation of glycolysis and tricarboxylic acid cycle pathways, thus drastically enhancing the flux of electrons along the mETC. Furthermore, advanced glycation end products (AGEs) [27], non-enzymatically glycated proteins [28], and insulin [29] all heighten ROS formation through the activation of NADPH oxidase; altogether, these factors act as major determinants of the exacerbated oxidative stress reported in the obese phenotype. 

In addition, hyperleptinemia (elevated plasma leptin levels) and obstructive sleep apnea (OSA, characterized by recurrent upper-airway obstructions caused by a loss of pharyngeal muscle tone during sleep), two processes intimately connected with obesity, may also upregulate the production of ROS via the activation of NADPH oxidase or after recurrent cycles of hypoxemia and reoxygenation, respectively [30]. At the hepatic level, obesity leads to a reorganization of mitochondria-associated ER membranes (MAMs) structures, triggering the overflow of calcium within the mitochondrion, eventually raising the intracellular levels of oxygen radicals [31]. 

Taken together, these observations lend further credibility to the hypothesis that oxidative stress and obesity stand in a relationship of mutual interdependency. In line with this finding, exacerbated oxidative damage has been implicated in the upregulation of sterol-regulatory-element-binding protein 1 (SREBP) and fatty acid synthase (FAS)-dependent lipogenic pathways, thereby worsening the obese condition [32].

Of note, it is plausible to speculate that ROS over production tied to obesity represents one of the major risk factors for the development of numerous obesity-related diseases such as diabetes, systemic arterial hypertension, ischemic heart diseases, liver failure or asthma.

## 3. Autophagy, between Obesity and Oxidative stress

### 3.1. Mechanism and Main Functions of Autophagy 

Autophagy, a term acquired from the Greek words “auto (self)” and “phagein (to eat)”, literally meaning “self-eating”, refers to an evolutionary conserved catabolic mechanism that allows cells to remove their own unnecessary or dysfunctional components [33]. This tightly regulated process underlies the sequestration of intracellular entities within double-membraned vesicles (called autophagosomes) and their incorporation into lysosomes for final degradation [34]. Autophagy can be classified into different subtypes, according to the modality of cargo delivery to the lysosome: macroautophagy (the main regulated form of autophagy that responds to environmental and physiological signals), microautophagy (i.e., the direct absorption of cytoplasmic contents by lysosomes), and chaperone-mediated autophagy (CMA; chaperone-assisted translocation of substrate proteins into the lysosome). It is worthy to note that macroautophagy (henceforth referred to as autophagy) can be further classified based on the material that is to be degraded [12] in non-selective autophagy (whereby the bulk part of the cytoplasm is catabolized in the autophagosome and recycled); or selective autophagy (whereby the material to be degraded is a specific substrate that undergoes receptor-mediated recognition prior to lysosomal delivery). In the latter setting, specific cargo includes protein aggregates (aggrephagy/proteophagy), endoplasmic reticulum (reticulophagy/ER-phagy), mitochondria (mitophagy), peroxisome (pexophagy), nucleus (nucleophagy), pathogens (xenophagy), lipids (lipophagy), or even lysosomes themselves (lysophagy) [12]. 

**Figure 2:**
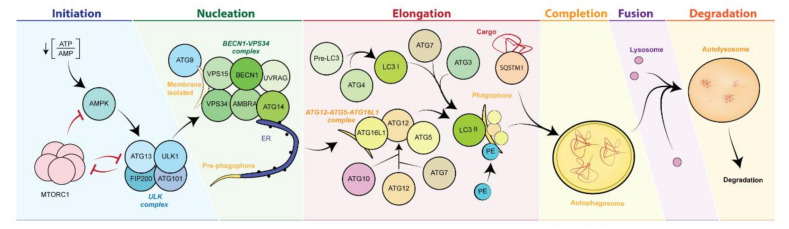
Molecular mechanisms of autophagy. In response to diverse stimuli, the activation of AMPK and/or the inhibition of mTORC1 stimulate the function of the ULK1 complex, which in turn activates the BECN1/VPS34 complex, starting the formation of the phagophore. Several ATG proteins catalyze the conjugation of the cargo adaptor LC3 to PE residues on the expanding phagophore membrane. Mature double membrane autophagosome loaded with its cargo eventually undergoes fusion with the lysosome, whereby its content is degraded and recycled. AMPK, 5′-adenosine monophosphate (AMP)-activated protein kinase; ATG, autophagy-related proteins; BECN1, beclin-1; CAV1, Caveolin 1, ER, endoplasmic reticulum; LC3, light chain of protein 1 associated to microtubules 3; MTOR, mechanistic target of rapamycin; PE, phosphatidylethanolamine; SQSTM1, sequestosome 1 protein (p62); ULK, UNC-51–like kinase.

At the molecular level, autophagy occurs through the spatiotemporal coordinated recruitment of specialized autophagy-related proteins (ATG) (the “core machinery”) and accessory proteins [35]. This process is tightly regulated and takes place in six sequential stages (Figure 2) [36]. (1) *Initiation*: The initiation of autophagy mainly relies on the action of the ULK complex, composed of the kinase 1 similar to unc-51 (ULK1), FIP200, ATG101, and ATG13 proteins. Importantly, the ULK complex constitutes a major regulatory hub of the entire autophagic pathway, as it receives and integrates positive and negative signals from upstream kinases. In direct concordance with this notion, the activity of the ULK1 complex is controlled by the delicate balance between activation inputs coming from 5′-adenosine monophosphate (AMP)-activated protein kinase (AMPK) [37] and inhibitory signals by mechanistic target of rapamycin (MTOR) complex 1 (mTORC1) [38], which both operate as key sensor of the energetic/metabolic state of the cell. On the one hand, AMPK responds to dwindling ATP levels (a recurrent condition under episodes of nutrients shortage) by inhibiting MTORC1 function or by directly promoting ULK1 activation, ultimately favoring autophagosome biogenesis. On the other hand, nutrient replenished conditions promote the activation of MTORC1, which keeps autophagy initiation at bay by inhibiting ULK1 and ATG13 [36,39].

(2) *Nucleation*: The pre-phagophore formation occurs through the activation of the BECN1/VPS34 complex (containing, among others, of the proteins VPS34 and BECN1), favored by ULK1 and AMPK1 kinase activity. [34,40,41,42]. (3) *Elongation*: The phagophore expansion is mainly controlled by ATG7 protein [43], which catalyzes the formation of the ATG12–ATG5–ATG16L1 complex (in concert with ATG10), and the conjugation of phosphatidylethanolamine with the light chain of protein 1 associated to microtubules 3 beta (MAP1LC3B; better known as LC3B) (in concert with the cysteine protease ATG4 and ATG3). The role of sequestosome 1 protein (SQSTM1; better known as p62), is essential for the binding and sequestration of autophagic substrates in the phagophore membrane [44,45,46]. (4) *Closure*: Once the cargo is bound to the phagophore membrane, the double-membraned structure closes (forming the complete autophagosome), in a process that depends upon ESCRT-0 and ESCRT-II [47]. (5) *Fusion*: The autophagosomal membrane fuses with the lysosomes, producing a new unilayered structure called autolysosome [48]. (6) *Degradation*: The autolysosomal cargo is digested within the acidic lysosomal environment, and the degraded material is released to the cytoplasm and eventually recycled for bioenergetics and structural purposes [49].

When executed at the baseline level, autophagy fulfills essential housekeeping duties and exerts a paramount function in the regulation of cellular homeostasis; of note, a significant surge in autophagy reportedly occurs under stressful conditions that include (but are not limited to) nutrients scarcity, organelle-specific damage, and invading pathogens. Regardless of the stressor, the stimulation of autophagy promotes cell survival by facilitating the return to the ground state. Based on these premises, it is hence not surprising that a proficient autophagic response is essential for maintaining the organism in a healthy status, while conferring upon it the ability to react (and adapt) to endogenous or exogenous insults. [50]. Conversely, settings of autophagy inhibition (e.g., mutations in ATG genes) map to accelerated ageing and directly correlate with the appearance of numerous age-associated diseases, such as CVD, metabolic disorders, neurodegenerative diseases, and cancer [51]. In the recent past, preclinical strategies based on the genetic or pharmacological activation of autophagy have held promises of therapeutic benefits in several human pathologies [52], in that they were shown to promote the extension of health-span and lifespan in several model organisms (including yeast, nematodes, flies, and mice) [53], thus positioning autophagy as a relevant clinical target in multiple disorders. 

### 3.2. Crosslink between Autophagy and Oxidative Stress

Pathological conditions (including obesity) characterized by exacerbated levels of oxidative stress exhibit a prominent raise in well-established markers of autophagy [54]. 

It hence appears plausible to speculate that autophagy induction takes active part to the general response orchestrated by the cell to counteract excessive ROS production. In this regard, among other functions, autophagy is essential to remove dysfunctional mitochondria, thus limiting the production of ROS under settings of nutrients overload [54]. While excessive intracellular ROS content is sufficient to generate a robust autophagic response [55], a transient increase in ROS production appears to be required to ignite autophagy upon scenarios of nutrient depletion [56] or exercise [57]. Accordingly, the administration of a ROS scavenger (e.g., N-acetylcysteine) inhibits the upregulation of autophagy that reportedly occurs under these conditions. 

**Figure 3:**
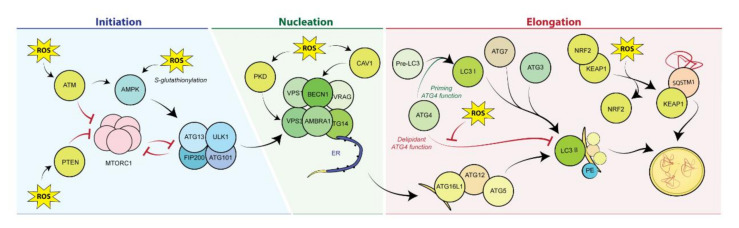
Reactive oxygen species–mediated autophagy induction. Autophagy is induced by ROS at different levels across the machinery, including initiation (through direct or indirect ROS-mediated modulation of AMPK and mTORC1 activity); nucleation, via Caveolin-1 or PKD-dependent activation of the VPS34/BECN1 complex and elongation, through ROS-dependent activation of Atg4. KEAP1. AMPK, 5′-adenosine monophosphate (AMP)-activated protein kinase; ATG, autophagy related proteins; ATM, ataxia-telangiectasia mutated; BECN1, beclin-1; ER, endoplasmic reticulum; KEAP1, Kelch ECH associating protein 1; LC3, light chain of protein 1 associated to microtubules 3; MTOR, mechanistic target of rapamycin; NRF2, nuclear factor erythroid 2-related factor 2; PE, phosphatidylethanolamine; PKD, protein kinase D; PTEN, phosphatase and tensin homolog; SQSTM1, sequestosome 1 protein (p62); ULK, UNC-51–like kinase.

The regulation of autophagy mediated by ROS is extensive and occurs at multiple levels throughout the autophagic circuit (Figure 3).

(1) Initiation: AMPK can upregulate the autophagic machinery under pro-oxidant conditions. H_2_O_2_ activates ataxia-telangiectasia mutated (ATM) in the cytoplasm, concomitantly leading to AMPK activation and mTOR repression [58]. Moreover, the oxidative-stress-induced S-glutathionylation of the α and β subunits of AMPK further potentiates its activity. Furthermore, elevated ROS levels account for the activation of phosphatase and tensin homolog (PTEN), which in turn restrains mTORC1 functions [59].

(2) Nucleation: Oxidative damage stimulates the activity of PKD, which phosphorylates VPS34 and promotes the autophagic reaction [60]. Similarly, settings of oxidative damage foster the pro-autophagic activity of the integral membrane protein Caveolin 1, which upregulates the autophagic flux via BECN1 phosphorylation [61]. 

(3) Elongation: ROS produced upon settings of nutrient scarcity are required for the oxidation of a cysteine residue within the catalytic domain of ATG4, which performs the enzymatic cleavage of LC3 [62]. 

(4) Other: Additional mediators of autophagy reaction are implicated in the response to oxidative stress, such as the forkhead transcriptional regulator FOXO1/FOXO3, c-Jun N-terminal kinase, or the endoplasmic reticulum kinase PERK [63].

Although the cause–effect nexus between ROS levels and autophagy induction is well established, the connections between these two processes are multidirectional. Impairment in the autophagic response is sufficient to heighten the burden of oxidative stress, which in turn contributes to deteriorate cellular functions. Furthermore, it has been observed that autophagy induction mediated by starvation, rapamycin [64], or physical exercise [57] produces a transient increase in ROS levels, favored by the enhanced mitochondrial activity associated with these experimental conditions. This result seems therefore to support the conjecture that transient bursts of stress (“hormesis”) may elicit positive effects at cellular and organismal level [65]. Reinforcing this finding, it is worthy to note that SQSTM1/p62 is not only involved in the recognition of the material to be degraded, yet it is also responsible for mounting an antioxidant response to counteract the increase in ROS levels that the autophagic process entails per se. Under conditions of autophagy induction, SQSTM1/p62 sequesters the E3 ligase Keap1, while preventing the proteasomal degradation of Nrf2; when released from the inhibitory liaison with Keap1, Nrf2 translocates to the nucleus, where it binds to antioxidant-sensitive elements (ARE) located in the promoter regions of multiple antioxidant genes [66], hence limiting oxidative damage.

### 3.3. Targeting Autophagy in Obesity

The pathogenesis of obesity underlies the prominent accumulation of potential autophagic substrates such as lipid droplets, protein aggregates, and damaged mitochondria. Therefore, an inhibition of autophagy can be expected to accelerate the development of obesity and its related pathologies. This over simplistic view may, however, be confuted based on the leading observation that a plethora of cell-intrinsic and cell-extrinsic factors are implicated in the etiogenesis and progression of this metabolic disorder. Therefore, an in-depth investigation regarding the specific role of autophagy in different compartments in vivo is warranted, in order to fully exploit its therapeutic potential in the prevention and treatment of obesity.

Overall, several evidences indicate that autophagy is repressed under obesogenic conditions. A steadily positive energetic balance fosters mTORC1 activity [67] at the expense of AMPK, achieving, as a net result, the inhibition of autophagy. In the liver (whereby autophagy is prominently activated under starvation) [68], long-term feeding of mice with a high-fat diet (HFD) is sufficient to stimulate mTORC1 activity and reduce the expression of ATG5 and ATG7 [69]. Along similar lines, genetic or pharmacological inhibition of autophagy counteracts fasting-induced weight loss, while contributing to the development of obesity and type II diabetes [70]. Consistent with this finding, chronic HFD feeding was shown to alter the intracellular ionic balance in hepatocytes, eventually hampering autophagosome–lysosome fusion [71,72]. Conversely, the liver-specific overexpression of Atg7 or Transcription Factor EB (TFEB) enhances autophagy, prevents weight gain, and alleviates signs of metabolic syndrome in both diet-induced and genetic mouse models of obesity [69,73]. Recently, the lipogenic protein Acyl-CoA-binding protein (ACBP) was found accumulated in the liver and the adipose tissue of mouse models of dietary or genetic induced obesity. ACBP is a protein secreted through an autophagy-dependent mechanism under conditions of nutrients scarcity. During starvation, autophagy allows ACBP to cross the plasma membrane and eventually reduces the levels of ACBP in the tissue. Defects in the autophagic process produce the aberrant accumulation of ACBP, and hence drive maladaptive lipogenesis [74]. Altogether, these results highlight autophagy as a key preventative process against the development of obesity-associated pathologies in the liver.

In sharp contrast to these observations, a significant upregulation in autophagy markers was reportedly described in type 2 diabetes specimens of human origin, potentially assigning the autophagic pathway a negative function in the progression of the disease [67]. Nonetheless, this interpretation is presumably biased by the fact that autophagy is monitored at the steady state, without accurate details regarding the autophagic flux [75].

Consistent with the anti-obesity activity reported in the liver, the pancreatic-beta-cell-specific inhibition of autophagy favors the establishment of a pro-diabetic phenotype, which is further aggravated by experimental settings of diet or genetic induced obesity [76].

In the recent past, a possible connection between autophagy and nutritional behaviors (i.e., food intake) has been put forward. The hypothalamic-directed suppression of autophagy (achieved through the delivery of a shRNA targeting Atg7 in the mediobasal hypothalamus) mapped to enhanced food intake, excessive weight gain, and defective energy expenditure [77]. In contrast to this datum, the genetic obliteration of Atg7 in AgRP hypothalamic neurons was sufficient to hinder the starvation–food intake feedback circuit, by favoring the appearance of a lean phenotype [78]. Further studies will be required to clarify the actual contribution of autophagy to the regulation of appetite.

Under specific circumstances, preclinical approaches based on the inhibition of autophagy may paradoxically contribute to mitigate some phenotypes associated with obesity [79,80]. Consistent with this notion, suppression of autophagy in hepatic stellate cells (HSCs) reduces CCL_4_-driven fibrotic scarring [81]; in addition, the autophagy dependent degradation of p62/SQSTM1 in HSC aggravates Diethynitrosamine (DEN)-mediated fibrosis, inasmuch as it limits the induction of an anti-fibrosis gene signature by Vitamin D Receptor (VDR): Retinoic Acid Receptor (RXR) heterodimers. 

Intriguingly, maneuvers that lead to the suppression of autophagy within the adipose tissue compartment may also elicit anti-obesogenic actions. It is worth noting that autophagy has been directly implicated in the interconversion of white adipose tissue (WAT, which mostly performs as an active site of lipogenesis) into brown adipose tissue (BAT, which operates as a central regulator of organismal thermogenesis by promoting the dissipation of chemical energy into heat through enhanced mitochondrial β oxidation). Upon adipose-specific deletion of Atg7, WAT acquires functional (but not transcriptional) features of BAT, including an increase in the mitochondrial content and oxidative metabolism [82]. Similarly, the suppression of autophagy in BAT improves energy usage, while limiting the obese phenotype [83]. Mechanistically, these effects can be attributed to the inhibition of mitophagy, which in turn restrains the WAT to BAT conversion while reinforcing BAT metabolic peculiarities.

In addition to the cell intrinsic effects listed above, circumstances of autophagy inhibition contribute to the obesogenic phenotype in a cell-extrinsic manner, inasmuch as they exacerbate inflammatory reactions [84]. While a functional autophagic response suppresses the inflammatory cascade, defects in autophagy are causally implicated in the production of inflammatory mediators. The suppression of autophagy favors the activation of NF-кB, which serves as a central transcriptional regulator of inflammation [85]. Furthermore, proficient autophagy accounts for the efficient lysosomal degradation of inflammasomes (e.g., NLRP3), supramolecular structures required for the caspase 1-dependent release of the pro-inflammatory cytokines IL-1β and IL-18 [84]. Along similar lines, the proper disposal of damaged mitochondria via autophagy restrains the activation of NRLP3 inflammasome, which is promoted by elevated intracellular ROS levels [86].

## 4. Treatments to Counteract Obesity-Associated Oxidative Stress

### Autophagy in Obesity-Associated Oxidative-Stress Therapies

Owing to the complex dialogue between oxidative stress and autophagy, and taking into account the multifaceted functions of this process within different cell types in vivo, disentangling the actual contribution of autophagy in anti-obesity therapies remains an open challenge.

It is noteworthy that the most effective treatments against obesity are well-known inducers of autophagy. These include lifestyle-based approaches (caloric-restriction-based strategies, low-calorie dietary regimens, and physical exercise), bariatric surgery, or pharmacological maneuvers [87]. In the latter scenario, compounds that have displayed anti-obesity properties include direct autophagy activators such as the mTORC1 inhibitor rapamycin [88], lipase inhibitors (e.g., orlistat), or appetite suppressants (e.g., phentermine or lorcaserin). Regardless of the exact mode of action, these agents exhibit the ability to alleviate excessive body-fat accumulation and to correct liver damage in preclinical models of alcoholic and nonalcoholic fatty liver by actively promoting lipolysis [74,89]. With the notable exception of lifestyle approaches, the implementation of the abovementioned pharmacological strategies in the clinical routine is often discouraged by the low benefit/risk ratio of these molecules [90], which makes their use not advisable. As previously mentioned, therapeutic avenues primarily based on the activation of autophagy may paradoxically exacerbate ROS production, potentially worsening the obese phenotype. Under such circumstances, alternative schedule regimens or treatment discontinuation (to be calibrated on the appearance of adverse reactions) may be adopted to overcome this issue. Alternatively, compounds endowed with antioxidant properties that do not compromise baseline autophagy can be used as preventative or curative measures against obesity. In keeping with this finding, pro-autophagic antioxidant supplements have been recently utilized to neutralize the detrimental effects of ROS in numerous pathological settings, including obesity. These encompass the following:

L-carnitine: L-carnitine is an amino acid derivative involved in lipids transport within the cell. In addition, it operates as free-radicals scavenger. Results from studies conducted in obese human patients revealed that L-carnitine supplementation elicited a significant impact on body-weight reduction [91]. L-carnitine stimulates autophagy and corrects high-fat-diet-induced mitochondrial dysfunction in the liver, while mitigating signs of obesity in mice [92].

Polyamines: Exogenous polyamines, such as spermine, spermidine and putrescine, are thought to mediate antioxidants’ functions in response to excessive ROS production, presumably acting as ROS scavengers [93]. Spermidine is contained at a high concentration in health-related products such as durian fruit, fermented soybeans, and wheat germs, and it is bona fide considered an efficient autophagy stimulator [94] with potent anti-obesity properties. Accordingly, the pharmacological stimulation of autophagy by spermidine reduces weight gain and improves obesity related parameters in mice [70].

Zinc: Zinc acts as cofactor for several antioxidant enzymes, but it can also function as an autophagy activator under circumstances of heightened oxidative stress, following its release from intracellular metallothionein [95]. Zinc supplementation for one month is sufficient to produce a significant decrease in body weight and body mass index in healthy obese human subjects [96].

Phenolic compounds: Examples of phenolic agents displaying both pro-autophagic and antioxidant properties include (among others) gallic acid and resveratrol. Treatment with gallic was shown to reduce excess body fat, curtail lipogenesis, and restrain inflammation in obese patients [97]. Likewise, long-term treatment with resveratrol showed protective effects against the development of the obese phenotype, while maximizing energy expenditure. Resveratrol stimulates autophagy through convergent modalities that include the activation of the AMPK–SIRT1–PGC-1α axis and the inhibition of the mTOR-ULK1 pathway [98], suggesting that autophagy may actually contribute to the beneficial effects of this agent in obesity. Nonetheless, the observation that resveratrol promotes WAT browning [99] indicates that additional mechanisms may underlie the pro-healthy actions of this molecule. 

While autophagy takes active part to the beneficial actions of these molecules in the prevention and treatment of obesity, it is important to note that these agents are often utilized at high concentrations, implying that they suffer from limited specificity. Accordingly, it is tempting to speculate that the positive effects of these molecules as anti-obesity treatment are tied to their ability to intercept multiple (rather than individual) cellular pathways. As a further element of complexity, agents that fall in the category of antioxidants and that display anti-obesity properties (such as Lipoic acid) inhibit (rather than promote) autophagy [100,101]. Therefore, additional studies are warranted to shed light on the hierarchy of events that account for the anti-obese functions of these molecules. Likewise, molecules known for their antioxidants properties (e.g., vitamins C and E) show no effect (or limited efficacy) when used as a standalone treatment against obesity.

## 5. Concluding Remarks

The extensive amount of works in the literature that were discussed in this review advocates for the hypothesis that suppression of autophagy, accompanied by an aberrant oxidative response, favors the establishment of an obesogenic environment. Nonetheless, given the multitude of factors involved in the pathogenesis of obesity, the contribution of autophagy to the obese phenotype varies in a context-dependent manner. With the advent of single cell technologies in the preclinical routine, it will be of primary importance to clarify the role of autophagy in different cell types (and pre-pathological settings) within a tissue, in order to pharmacologically harness this process in the anti-obesity therapy.
